# Effects of an Information and Communication Technology-Based Fitness Program on Strength and Balance in Female Home Care Service Users

**DOI:** 10.3390/ijerph18157955

**Published:** 2021-07-28

**Authors:** Sonja Jungreitmayr, Susanne Ring-Dimitriou, Birgit Trukeschitz, Siegfried Eisenberg, Cornelia Schneider

**Affiliations:** 1 Department of Sport and Exercise Science, Paris Lodron University of Salzburg, 5400 Hallein-Rif, Austria; susanne.ring@sbg.ac.at; 2 Research Institute for Economics of Aging, Vienna University of Economics and Business, 1020 Vienna, Austria; Birgit.Trukeschitz@wu.ac.at (B.T.); siegfried.eisenberg@gmail.com (S.E.); 3 Institute of Computer Science, University of Applied Sciences Wiener Neustadt, 2700 Wiener Neustadt, Austria; Cornelia.Schneider@fhwn.ac.at

**Keywords:** exercise, function/mobility, assistive technology, assisted living

## Abstract

There is evidence that training for strength and balance prevents decline in physical function in old age when the training is personally instructed. It is an open question whether interventions that deliver training via up-to-date technologies can achieve long-term effects. This study examined the effects of an 8-month fitness training program delivered via information and communication technology (ICT) on lower-body strength and balance in female home care users (*n =* 72) aged 75 years on average. For statistical analysis, the test group was divided into two subgroups, one who used the program at least 8 times per month (*n =* 26) and another one who used the program less often (*n =* 17) compared with a control group that received no exercise program (*n =* 29). It was found that regular ICT-exercisers exhibited positive effects over time on lower-body strength and balance compared to a decrease in both indicators in irregular exercisers and the control group. The authors see potential in offering exercise programs to people of advanced age via ICT to counteract physical decline in old age.

## 1. Introduction

Staying functionally fit, which means having the physical capacity to perform motor tasks safely, independently, without excessive stress in everyday life, is vital for independent living, especially among older adults [1]. Exercise programs that address different components of physical fitness, such as strength as well as balance, prevent the deterioration of physical functions in old age [2]. Evidence-based general recommendations on physical activity (PA) for older adults suggest integrating regular endurance, strengthening, balance, and flexibility exercises into the weekly exercise program, whereas training for strength should be completed at least twice per week [3,4,5,6].

Most interventions that promote physical activity in older adults are carried out under personal supervision and guidance, which ties up a large number of resources per case [7]. However, there is evidence that positive effects on strength and balance can be achieved when movement interventions are carried out without supervision [8]. Modern technologies can easily transport unsupervised training and are becoming more available, creating new opportunities in terms of reducing costs and economic burden and improving the quality of life for people as they age [7,9].

While the current literature finds positive effects in short-term information and communication technology (ICT)-based interventions to promote physical activity in people between 55 and 80 years of age [7], there is a need for further research in specific user groups regarding long-term effects using state-of-the-art technologies. One user group growing larger is that of older persons in need of assistance. According to recent WHO data, 14% of all people over the age of 60 lack functional ability in daily living [10]. A closer look at home care service users (HCSU) shows that, within this population, women live longer with impaired quality of life than men [11]. Gender differences are also noticeable, with women needing more assistance with activities of daily living in later life than men, due to their lack of strength [8]. Women within this population are also more likely to fear falling, which is associated with impaired balance ability [12]. Because both strength and balance ability are critical for independent living later in life in women, the effects of novel training on these markers should be specifically investigated.

There is thus evidence that ICT-assisted training can promote physical activity in older people in general [7] and that this is particularly important in women [11]. Furthermore, there are also recommendations to use comprehensive devices for promoting physical activity [13]. Likewise, it can be considered certain that supervised functional training can have positive effects on physical status in older women [12,14,15,16]. Nevertheless, over the long term, to the best of our knowledge, there is still no evidence whether unsupervised functional training delivered via ICT has a positive effect on the development of strength and balance in older women with mild assistance needs.

Therefore, the purpose of this study was to examine the effects of an ICT-guided functional fitness program on strength and balance of female HCSUs who were on average 75 years old, over eight months, and to analyze whether following the recommended exercise frequency makes a difference. In this context, we hypothesize that unsupervised, functional ICT-supported fitness training will have positive effects on strength and balance outcomes in female HCSUs when performed at least twice a week, compared to lower usage or no access to the ICT-program at all.

## 2. Materials and Methods

### 2.1. Study Design

The group studied in this paper is the subset of female HCSUs targeted in the CiM project (Care-in-Movement), which aimed to develop an ICT-enabled care support system for HCSUs and their environment. In this project, a parallel group design with matched pairs was conducted on male and female HCSUs in Austria and Northern Italy [17]. This design is a very focused recruitment method that closely resembles a fully randomized controlled trial design [18]. From an available data set of participants who met predefined eligibility criteria (age between 55 and 85 years, no cognitive impairments, no more than low limitations in hearing and/or vision, and/or defined limitations in mobility), individuals were randomly selected for the intervention group and matched for the control group. The recruitment started after this preparation. In order to comply exactly with all the required specifications, the recruiters were obliged to follow a detailed protocol [17]. 

HCSUs assigned to the test group (TG) received an ICT-based intervention. The intervention consisted of a functional fitness program (FFP) with daily alternating 10-min training sessions delivered via a specially designed tablet application and lasted 8 months [19]. The participants assigned to the control group (CG) had no access to the ICT-based intervention. 

### 2.2. Data Collection

All study participants in both countries were offered to be tested three times in their homes by their caregivers, at baseline (t0), after 6 months (t1), and at the end (t2) of the trial period. The test at t1 was scheduled after six months to ensure a possible change in physical activity level [20], and thus to expect effects on strength and balance. The final test (t2) was conducted to ensure impact analysis over the full eight-month period. A total of 109 female HSCUs showed up on all three occasions.

The care workers collected data with standardized equipment. System usage data needed for grouping and adherence evaluation were collected by a logging component (Matomo, InnoCraft, 150 Willis St., Wellington, New Zealand) [19].

### 2.3. Intervention: ICT-Supported Functional Fitness Program (FFP)

After the experts had assigned an appropriate exercise difficulty and intensity level, the software compiled an order of exercises based on the workout structure (see Table 1). For this purpose, the exercises were stored in a hidden library within the tablet and sorted by category. The “Joint Mobility Exercises” category contained simple movements such as arm circles, which involved moving through the joints without loading. Exercises for the upper extremity and spine joints were labeled “Joint Mobility Exercises 1,” and those for the lower extremity and spine were labeled “Joint Mobility Exercises 2.” The “Coordination Exercises” consisted of complex movements—such as circling the arms while marching on the spot. The “Balance Exercises” included variations of the one-legged stand such as leg-swings and tandem stand, with or without holding onto the back of a chair, depending on the exercise difficulty selected. Finally, the category “Strengthening Exercises” included motor tasks that stressed the entire body or upper body (Strengthening Exercises 1) and the lower body (Strengthening Exercises 2), such as table push-ups, extending the knees while sitting, lifting the heels while standing, lunges with or without holding on to a chair back. The exercises in the training sessions were delivered visually, with short video clips, and vocally, with simple explanatory text in the local language via the tablet app. To maintain adherence of ICT-program users, the exercises changed daily, providing variety and attractiveness. The training sessions were kept as short as possible, lasting on average 10 min, following the general recommendations [3,4,5]. Every session consisted of six bodyweight-bearing exercises (see Table 1). 

After a warm-up phase containing joint mobility exercises, the main part of each session included exercises for both coordination as well as strength. The volume for each coordination exercise was set at two sets for 40 s per set [21]. Strength training was set at two sets per exercise in a repetition range from 8 to 12 repetitions per set to initiate adaptations [22]. This methodology ensured that people who chose to do the exercise program at least two times a week would have reached the minimum dose for a neuromotor stimulus for increased balance as well as strength [23].

### 2.4. Measures

#### 2.4.1. Anthropometric Data

Body height in m and body mass in kg were measured both accurate to a tenth with a mobile stadiometer (Seca 213, Seca GmbH, Hamburg, Germany) and a digital scale (Smartlab scale W, HMM Diagnostics GmbH; Heddesheim, Germany) according to standardized procedures [24]. Body Mass Index (BMI = kg/m^2^) was calculated as body mass in kilograms divided by height in squared meters, again accurate to a tenth [24].

#### 2.4.2. Overall Strength—Grip Strength (GRIP)

Grip strength, which is a useful tool for screening older adults for risk of future health deterioration as well as a potent surrogate measure for overall strength, was tested with a hand-dynamometer (Deyard EH101, Deyard Technology Ltd., Shenzhen, China) [25,26].

HSCUs were asked to sit upright on a chair, both feet planted on the ground, hip-width apart, one arm hanging loosely on the body side, the other arm positioned on a desk plate, bent at 90 degrees. The hand-dynamometer was then placed in a neutral grip position into the hand, resting on the counter. Then, the handle had to be squeezed without changing posture. The assessment was conducted in an alternating fashion for a total of three repetitions per hand. The mean of all repetitions from both sides was calculated. Results were measured to the nearest tenth of a kilo [27].

#### 2.4.3. Lower-Body Strength—30s Chair Rise (30CR)

The 30 s Chair Rise test indicates lower-body strength among older community-dwelling adults [28]. All trial participants were asked to sit upright on a chair without using the backrest. Foot position had to be at hip-width. Arms were crossed over the chest with the hands touching the contralateral shoulder. The proper execution of the task was examined before starting the testing procedure. After checking for readiness, the command 3-2-1-go was applied, and the completed repetitions within 30 s were recorded. If the participant ended up standing at the lapse of the 30 s timeframe, this was counted as 0.5 repetitions. The results were recorded to the nearest half repetition.

#### 2.4.4. Balance—Uni-Pedal-Stance (UPS)

UPS is a valid measure of static balance [29]. All trial participants were asked to place the hands on their hips and focus on an imaginary spot on the eye-level at the wall, right opposite to them. The test ended when the position was exited via (1) lowering the lifted leg onto the ground, (2) twisting the ground-based foot to stay in balance, (3) lifting the arms off the hips, and (4) pressing the lifted leg onto the ground-based leg or if the person could stand one-legged for over 60 s. This was applied in alternating fashion up to six trials in total, three for each leg. The best score of all trials of both legs was included in the evaluation to the nearest tenth of a second.

### 2.5. Sample 

**Figure 1:**
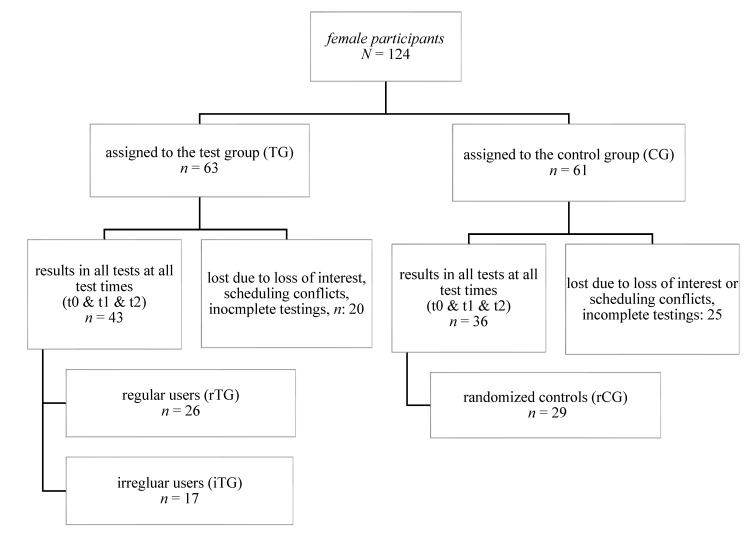
Flow of Participants Regarding Division Into Evaluation Groups/regular users used the system > 8 times per month, irregular users used it less than 8 times per month.

Female participants enrolled in CiM (*N* = 124) provided the basis for our evaluation. Female participants who participated in all tests at each time point (t0, t1, t2) were included in this data analysis (see Figure 1) and further subdivided by adherence for statistical analyses. 

Adherence is defined here as active participation in the intervention program. To measure this participation, automatically logged usage data were used [19]. Usage data gives insights about which components of the app were used, how often they have been used and when they have been used. Schneider (2020) utilized this information to build sub-groups based on the usage frequency of the system. The smallest unit in this measuring framework was a “visit”. A “visit” started with the first use of the system on a new day or after a pause of at least 30 min. To be counted as “adhered to” an exercise screen had to be on for 30 s or longer. This timespan was based on the time used for one training set (40 s or 8 to 12 reps; see Table 1). User groups were then formed, based on visits that contained adhered exercises. Frequent users did use the training program at least 8 times per months, regular users between 4 and 7 times per month, infrequent users adhered between 1 and 3 times per months and people who never used the exercise program were grouped into non-users [19].

Since the minimum dose for a neuromotor stimulus to increase balance as well as lower body strength is two times per week [23], we recombined these groupings for the study of effects on markers of strength, and balance. The rTG included all subjects who were classified as regular users in the usage data analysis, i.e., who had used the program at least eight times per month and thus showed sufficient adherence. The remaining groups were combined as the irregular test group (iTG), as these usage frequencies do not indicate sufficient adherence. To avoid low power due to unequal sample sizes, we selected *n* = 29 participants of the CG (rCG) by the random number function in SPSS for the comparison between sub-groups [30].

### 2.6. Statistical Analysis

Statistical analysis was carried out using SPSS (version 27.0; IBM Corp., Armonk, NY, USA). Baseline data were described with mean ± standard deviation. To check whether anthropometry was an influencing factor of the outcome variables and thus had to be considered as a covariate, a correlation analysis, using Pearson’s correlation coefficient *r*, was calculated [31].

To evaluate the effects over time and between groups, the first step was to test for normal distribution using Shapiro—Wilk’s test. Normally distributed data, which also did not violate Levene’s test for equality of variances, were analyzed by analysis of variance (ANOVA) to compare the characteristics of the subgroups with respect to these variables, at each test date. If significant differences between subgroups were found, pairwise comparisons were performed using the Scheffe procedure [32]. To account for possible baseline differences in the effects between groups and over time, an ANCOVA adjusted for baseline values, was calculated.

Nonparametric methods were chosen to examine the effects between groups and over time of data that did not follow the normal distribution assessed by the Shapiro–Wilk test or violate Levene’s test for equality of error variances. The Kruskal–Wallis H test was executed to assess differences at times between subgroups [33]. Changes over time within subgroups were analyzed via the Friedman test, respectively [34]. Results were reported accordingly, with median or median rank values. If statistically significant differences between subgroups were detected, pairwise comparisons using Dunn’s procedure with a Bonferroni correction for multiple comparisons were performed [35]. After performing the nonparametric test, a repeated measures nonparametric analysis of covariance (ANCOVA) was run [36]. As groups vary in size, the Scheffe procedure was chosen to serve as post hoc test [32].

The level of significance was set to *p* < 0.05. Adjusted *p*-values are presented. Effect sizes are expressed as Pearson’s *r* in the Kruskal–Wallis H as well as the Friedman test and as ηp^2^ in ANOVA as well as ANCOVA, whereas *r* is considered to be a relatively small effect at 0.1, typical at 0.2 and relatively large at 0.3 [37] and ηp^2^ is defined to be a small effect at 0.01, moderate at 0.06 and large at 0.14 [38]. 

## 3. Results

### 3.1. Basline Data

Table 2 shows baseline characteristics of subgroups in descriptive measures. On average, the female HCSUs were aged 75.5 (±7.6) years (*n* = 72) with a mean BMI of 29.2 (±6.4) thus should be categorized as overweight to obese [39]. Bonferroni post hoc analysis revealed that the iTG had higher BMI + 4.9, 95%CI (3,9.5) than rTG, which was statistically significant (*p* = 0.034) as well as rCG + 5.5, 95% CI (1.0, 10.0) which also was a significant difference (*p* = 0.012), but no other group differences were statistically significant regarding BMI. Given that BMI is a potential confounder of the functional markers [40,41], the correlation of BMI with the outcome variables was calculated. Since Pearson’s *r* showed no correlations between BMI and outcome variables at all test time points, BMI was not included as a covariate in follow-up calculations.

**Figure 2:**
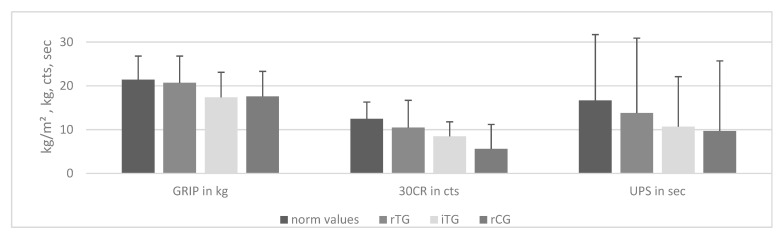
Comparison of Baseline Results With Gender- and Age-Specific Norm Values/This figure shows the baseline data of the subgroups compared to correspondingly matching norm values. GRIP = grip strength; 30CR = 30 s Chair Rise test; UPS = Uni Pedal-Stance. Data presented as mean (incl. SD one-sided) in kg = kilogram, cts = counts or sec = seconds. rTG = regular users; iTG = irregular users; rCG = randomized controls.

As Table 2 and Figure 2 show, the results of the first measurement indicate insufficient or poor fitness of the participants compared to normative values in terms of grip strength, lower-body strength, and static balance ability regardless of subgroup [29,42,43]. This means that the trial participants started at a below average functional fitness level.

### 3.2. Adherence

A total of 124 females attended at least one of three tests (t0, t1, and t2). Forty-three females belonged to the TG, received the intervention, and were present at all tests. Thirty-six females belonged to the CG and were present at all tests (see Figure 1). The TG was, as stated before, divided into regular (rTG; usage rate ≥ 8x/month) and irregular (iTG; usage rate < 8x/month) users. 60% of the TG who attended all three test sessions used the system regularly in this way and could be attributed to the rTG. The remaining TG participants who were present at all test sessions used the system 1 time per week or less, i.e., irregularly, and were therefore assigned to the iTG.

### 3.3. Effects on Grip Strength Measured by GRIP

While there is no significant difference within groups regardless of subgroup as well as between groups at baseline and t2, ANOVA shows a highly significant difference between groups at t1 (see Table 3). 

Pairwise comparisons found the difference in t1 to be between rTG and iTG at *p* = 0.036 (0.25; 9.0) as well as between rTG and rCG at *p* = 0.025, 95% CI (0.44; 8.0).

**Figure 3:**
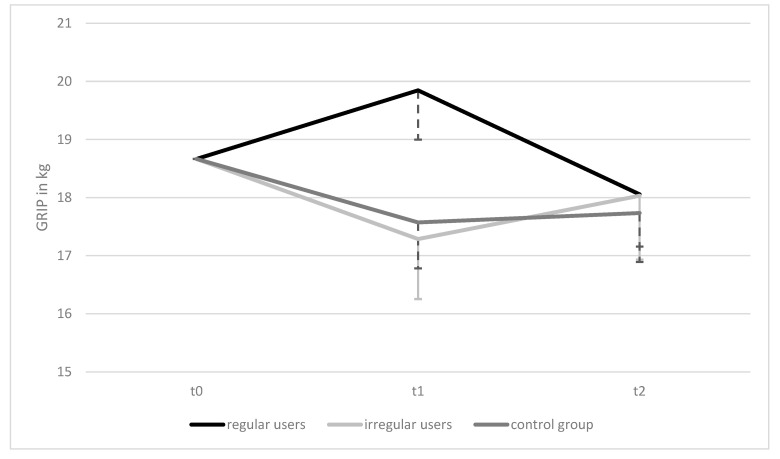
Mean Differences in GRIP ANCOVA Results, Over Time and Between Groups/GRIP = mean grip strength of all attempts from both hands. Data presented as means of all attempts per test date on all three test dates adjusted for baseline values. Error bars (one-sided): 95% CI.

Two-way ANCOVA (adjusted to baseline scores) with repeated measures showed that the intervention elicited no statistically significant changes in GRIP within groups over time, *F*(4, 136) = 1.554, *p* = 0.192, ηp^2^ = 0.044 and also no significant differences between groups over time, *F*(2,68) = 0.858, *p* = 0.429, ηp^2^ = 0.025, but clearly displays the difference at t1 found in the ANOVA (see Figure 3).

### 3.4. Effects on Lower-Body Strength Measured by 30CR

The Friedman test revealed no significant changes within groups in 30CR over the course of the intervention regardless of which group is considered. 

Significant differences between groups in median scores were found at all times via Kruskal—Wallis H test and post hoc analysis revealed statistically significant differences in 30CR between rTG and rCG at all times (see Table 4). 

Pairwise comparisons revealed significant differences between rTG and rCG at baseline (*p* = 0.008, *r* = 0.41), as well as at t1 (*p* = 0.034, *r* = 0.34) and at t2 (*p* = 0.001, *r* = 0.50), but no other differences between groups.

**Figure 4:**
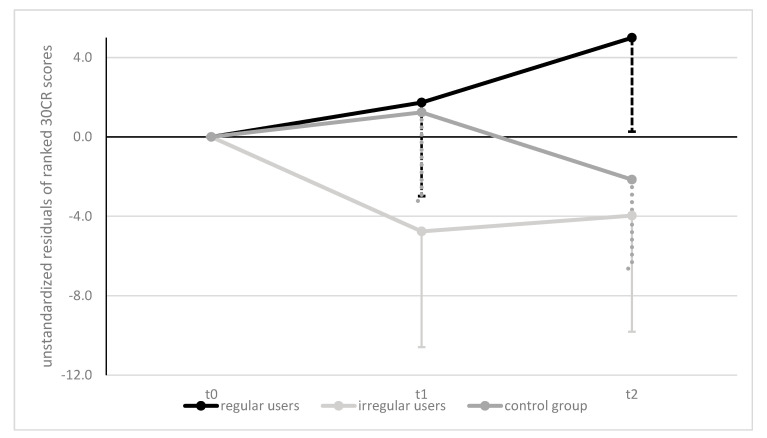
Mean Differences in 30CR ANCOVA Results, Over Time and Between Groups/30CR = 30 Seconds Chair Rise test. Data presented as unstandardized residuals of ranked scores on all three test dates adjusted for baseline scores. Error bars (one-sided): 95% CI.

Non-Parametric two-way ANCOVA (corrected for baseline scores) with repeated measures showed that the intervention elicited statistically significant changes in 30CR scores within groups over time, *F*(4, 138) = 2.695, *p* = 0.033, ηp^2^ = 0.072 and a trend for differences between groups over time, *F*(2,69) = 2.658, *p* = 0.077, ηp^2^ = 0.072 (see Figure 4). Post hoc testing showed a significant mean difference between rTG and iTG at *p* = 0.008 but no other effects.

### 3.5. Effects on Balance Measured by UPS

Analysis of differences within groups over time, via Friedman test, showed significant differences in rTG, χ^2^(2) = 6.320, *p* = 0.042, and iTG, χ^2^(2) = 10.082, *p* = 0.06, but not in rCG, χ^2^(2) = 0.840, *p* = 0.657, whereas pairwise comparisons revealed the drop from t0 to t1 scores in iTG being statistically significant (*p_adj_* = 0.024, *r* = 0.22).

Kruskal—Wallis testing showed a significant difference at t1 between groups (see Table 5). Pairwise comparisons revealed significant differences between rTG and rCG at t1 (*p* = 0.017, *r* = 0.37), but no other differences at times between groups.

**Figure 5:**
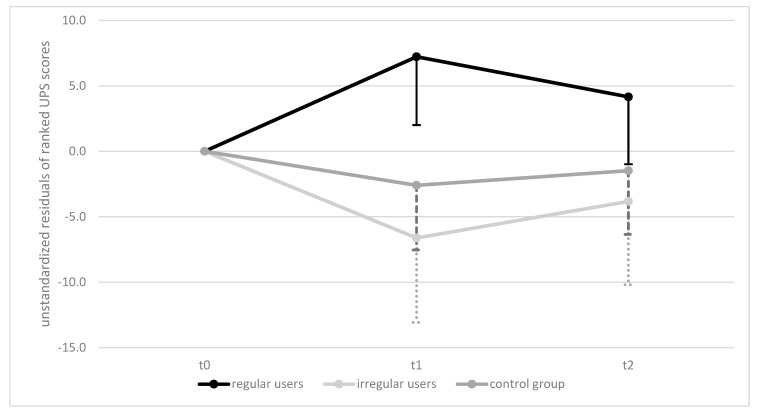
Mean Differences in UPS ANCOVA Results, Over Time and Between Groups/UPS = Uni Pedal Stance. Data presented as unstandardized residuals of ranked UPS scores on all three test dates adjusted for baseline scores. Error bars (one-sided): 95% CI.

Figure 5 presents results of the nonparametric two-way ANCOVA (corrected for baseline scores) with repeated measures. The analysis showed that the intervention elicited statistically significant changes in UPS scores within groups over time, *F*(4, 138) = 4.506, *p* = 0.008, ηp^2^ = 0.116 and also for differences between groups over time, *F*(2,69) = 4.279, *p* = 0.018, ηp^2^ = 0.110. Post hoc testing showed a significant difference between rTG and iTG at *p* = 0.03 and a trend towards difference between rTG and rCG at *p* = 0.093.

## 4. Discussion

Supporting older people, especially HCSU, to prevent physical decline [44] is more important than ever. It is especially important for women who are already in need of care to stay active, as they have lower fitness levels than their independently living peers and their abilities decline sharply without physical engagement [45]. As technological capabilities continue to increase, it is necessary to consider whether they can be used as potent tools for keeping HCSU fit. The objective of the study was to determine whether an unsupervised, functional ICT-mediated exercise program, with a minimum use of two times per week, has positive effects on the development of strength and balance in older, female HCSUs, compared with those who use the program less frequently or do not have access to the program. The data show that the studied training program increased fitness in regular users, which is in line with the outcome of other ICT-exercise programs [46].

Sixty percent of the here-investigated TG used the FFP at least eight times per month, according to the usage evaluation [19]. Compared with other eHealth interventions, which are typically designed for once-a-week use and achieved adherence of about 50%, this intervention exhibited a remarkably high adherence rate [47]. In terms of adherence rate, our intervention was also superior to a comparable supervised program consisting of 32 one-hour sessions over six months, where only 19 of 40 subjects (48%) completed a sufficient number of sessions, indicating a lower adherence rate compared with our ICT program [48]. However, a low adherence rate is not unusual. It is estimated that exercise programs for older people have a drop-out rate of about half within the first 6 months [49] and adherence is typically reported to be poor for exercise programs [50]. The brevity of the training sessions and the strength training components could be reasons for this good adherence rate. The latter was found to elicit higher adherence rates compared to endurance training in general [51]. Moreover, it could also speak for the design of both the user interface of the app as well as the intervention itself. Our data show that a non-supervised exercise program can achieve adequate adherence rates among female HCSUs when delivered via ICT and thus can have an influence on physical fitness.

Grip strength, which serves as a surrogate for overall strength [26] was affected by our intervention especially over the first period. The results of the rTG at t1 point towards better strength of the subjects who used the application regularly compared to the iTG and CG. The reason that this effect did not last until the end of the trial may be attributed to the fact that due to the brevity of the sessions, the program focused on lower-body strengthening as well as balance, thus upper-body dominant exercises were less represented. It is likely that this parameter would have needed a more extensive training program to experience sustained increases. This finding is consistent with the results of the Lifestyle Interventions and Independence for Elders (LIFE) study [52], which assessed grip strength at 6-month intervals over a period of 36 months, among other measures. Although markers such as the Short Physical Performance Battery (SPPB) score or the 30 s chair rise test were improved, there was no increase in grip strength measured either. Similar to our study, this was attributed to the focus of the training program, which addressed lower extremities.

Lower-body strength expressed as 30 s chair rise ability increased in those who used the FFP regularly and decreased with low usage or in controls. This finding is consistent with existing results demonstrating that lower-body strength, measured via 30 s chair rise in care-using women, significantly decreases over 8 months due to insufficient activity without intervention [45]. Our data underline the positive effect of the program on the lower-body strength of those who have used the application at least twice a week (see Figure 3). These results fit well with the recommendations for health-promoting physical activity, which recommend total body strengthening at least twice a week [3]. It has already been studied, that implementing these recommendations in a supervised setting leads to more lower-body strength in care-users [16] as well as in community-dwelling adults [45,52]. Now we have preliminary evidence that improvement of this parameter is also possible with ICT-mediated training programs.

As balance can be seen as a potent precursor of improvements in other motor abilities, the increase of 30CR scores may be attributed to improved balance ability [53,54]. Static balance ability increased in the first six months of intervention and dropped slightly above baseline within the rTG (see Figure 4), which is consistent with findings where an improvement was achieved within the first few months, followed by a slight reduction in balance performance in the subsequent months [52]. However, in our study, the values dropped significantly in those who started at reasonably good levels (iTG) but did not use the system regularly. In CG, which began at an extremely poor level, the deterioration in balance ability progressed to the point where at least half of the group could not stand on one leg for a single second. Thus, the training program studied had positive effects on balance over 8 months as it was able to counteract degradation in balance performance.

In light of these findings, we emphasize the importance of regular physical training for female HCSUs, highlighting that even short training sessions can produce positive effects for this group of individuals, and note that this can be achieved by an unsupervised ICT-mediated fitness program. The strength of our study is that we measured the effects under normal field conditions [55] with valid indicators for all outcomes [25,26,28,29] and a good adherence rate over a long-term course of eight months. Participants who regularly followed the FFP maintained their strength and balance at baseline levels or showed improvements compared to their peers. Despite similar baseline levels, participants who did not follow the FFP regularly showed a decrease in bodily functions over the course of eight months that was similar to the trends in the control group.

Due to the small sample size, a problem that is often the case in such study settings [46], the results should be treated with caution with regard to their generalizability. The study was not completely randomized because HCSUs had the right to decline assignment to any of the groups, which correspondingly affects the external validity of the results. The automatically logged usage data provide insight into time and frequency of use, without evidence of actual exercise performance or an assessment of exercise quality.

## 5. Conclusions

In conclusion, the ICT-assisted fitness training examined in this study can be well applied to counteract losses in strength and balance in female HCSUs aged 60 to 91 years with low support needs. Adherence rates indicate that this new way of training is well accepted by females of age who want to maintain their physical condition, when designed to their needs.

## Figures and Tables

**Table 1:** Order of Main Motor Abilities Addressed in a 10-min Workout Routine.

Workout Routine—Structure			
WarmUp	Joint Mobility Exercise 1	Set 1	40 s
	Joint Mobility Exercise 2	Set 1	40 s
	Joint Mobility Exercise 1	Set 2	40 s
	Joint Mobility Exercise 2	Set 2	40 s
Coordination	Coordination Exercise	Set 1	40 s or 8–12 reps
	Balance Exercise	Set 1	40 s
	Coordination Exercise	Set 2	40 s or 8–12 reps
	Balance Exercise	Set 2	40 s
Strength	Strengthening Exercise 1	Set 1	8–12 reps
	Strengthening Exercise 2	Set 1	8–12 reps
	Strengthening Exercise 1	Set 2	8–12 reps
	Strengthening Exercise 2	Set 2	8–12 reps

Note. This figure shows the structure of a 10 min session. Duration per exercise is indicated either in seconds (=s) or repetitions (=reps).

**Table 2:** Baseline Characteristics of Sub-Groups.

Descriptives	rTG		iTG		rCG	
	*n* = 26		*n* = 17		*n* = 29	
	*M*	*SD*	*M*	*SD*	*M*	*SD*
Age, yrs	74.4	6.8	75.5	7.4	76.6	8.5
Height, cm	158.4	7.7	159.2	7.5	160.8	6.3
Weight, kg	71.7	18.5	84.5	21.3	71.5	15.6
BMI, kg/m²	28.3	5.1	33.2	7.3	27.7	6.0
GRIP, kg	20.7	6.1	17.4	5.7	17.6	5.7
30CR, cts	10.5	6.2	8.5	3.3	5.6	5.6
UPS, s	13.8	17.1	10.7	11.4	9.7	16.0

Note. rTG = regular users; iTG = irregular users; rCG = randomized controls.

**Table 3:** Differences in GRIP Between Groups at Times.

GRIP, kg	rTG		iTG		rCG		*ANOVA*		
	*n* = 26		*n* = 17		*n* = 29				
	*M*	*SD*	*M*	*SD*	*M*	*SD*	*F* (2/69)	*p*	*η* ^2^
t0_GRIP_mean	20.7	6.1	17.4	5.7	17.6	5.7	2.411	0.097	0.065
t1_GRIP_mean	21.1	6.6	16.5	5.7	16.9	4.6	5.088	0.009	0.129
t2_GRIP_mean	19.3	6.4	17.2	4.8	17.0	5.9	1.218	0.302	0.034

Note. rTG = regular users; iTG = irregular users; rCG = randomized controls; t0_GRIP_mean = mean grip strength of all attempts from both hands at first test date. t1_GRIP_mean and t2_GRIP_mean 30CR correspondingly results of subsequent tests. Data presented as kilogram to the nearest tenth. *p* = 0.05.

**Table 4:** Differences in 30CR Between Groups at Times.

30CR, cts	rTG	iTG	rCG	*Kruskal–Wallis*	
	*n* = 26	*n* = 17	*n* = 29		
	*Mdn*	*Mdn*	*Mdn*	χ^2^ (2)	*p_adj_*
t0_30CR	10.5	10.0	5.0	9.580	0.008
t1_30CR	12.0	9.0	7.0	6.666	0.036
t2_30CR	13.5	10.0	5.0	13.645	0.001

Note. t0_30CR = results of the 30 Seconds Chair Rise test on the first test date; t1_30CR and t2_30CR correspondingly results of subsequent tests. rTG = regular users; iTG = irregular users; rCG = randomized controls. Data presented as counts. *p* < 0.05.

**Table 5:** Differences in UPS Between Groups at Times.

UPS, sec	rTG	iTG	rCG	*Kruskal–Wallis*	
	*n* = 26	*n* = 17	*n* = 29		
	*Mdn*	*Mdn*	*Mdn*	χ^2^ (2)	*p_adj_*
t0_UPS	5.8	8.0	0.0	2.604	0.272
t1_UPS	15.0	2.0	0.0	8.589	0.014
t2_UPS	11.0	8.0	0.0	4.290	0.117

Note. t0_UPS = results of the Unipedal stance test on the first test date; t1_UPS and t2_UPS correspondingly results of subsequent tests; rTG = regular users; iTG = irregular users; rCG = randomized controls. Data presented as counts. *p* < 0.05.

## Data Availability

The data presented in this study are available on request from the corresponding author. The data are not publicly available due to privacy.
